# Baseline assessment of pharmacovigilance activities in four sub-Saharan African countries: a perspective on tuberculosis

**DOI:** 10.1186/s12913-021-07043-6

**Published:** 2021-10-08

**Authors:** Everdina W. Tiemersma, Ibrahim Ali, Asnakech Alemu, Yohanna Kambai Avong, Alemayehu Duga, Cassandra Elagbaje, Ambrose Isah, Alexander Kay, Blandina Theophil Mmbaga, Elice Mmari, Kissa Mwamwitwa, Siphesihle Nhlabatsi, Kassech Sintayehu, Aida Arefayne, Mekonnen Teferi, Frank Cobelens, Linda Härmark

**Affiliations:** 1 Technical Division, KNCV Tuberculosis Foundation, PO Box 146, 2501 CC Den Haag, The Netherlands; 2 National Pharmacovigilance Centre, Pharmacovigilance/Post Marketing Surveillance Directorate, National Agency for Food and Drug Administration and Control, Abuja, Nigeria; 3 Ethiopia Food and Drug Authority, Addis Ababa, Ethiopia; 4 Institute of Human Virology Nigeria, Federal Capital Territory, Abudja, Nigeria; 5 University of Technology Sydney, Sydney, New South Wales Australia; 6 Children’s Foundation, Baylor College of Medicine, Mbabane, Eswatini; 7 National Pharmacovigilance Center, Ministry of Health, Matsapha, Eswatini; 8 KNCV Tuberculosis Foundation, Abudja, Nigeria; 9 Department of Clinical Pharmacology and Therapeutics, School of Medicine, College of Medical Sciences, University of Benin, Benin, Nigeria; 10 Department of Pediatrics, Baylor College of Medicine, Houston, USA; 11 Baylor Children’s Foundation-Eswatini, Mbabane, Eswatini; 12 Kilimanjaro Clinical Research Institute, Moshi, Tanzania; 13 Kilimanjaro Christian Medical University College, Moshi, Tanzania; 14 KNCV Tuberculosis Foundation, Dar es Salaam, Tanzania; 15 Tanzania Medicines and Medical Devices Authority (TMDA), Dar es Salaam, Tanzania; 16 KNCV Tuberculosis Foundation, Addis Ababa, Ethiopia; 17 Clinical Trials Unit, Armauer Hansen Research Institute (AHRI), Addis Ababa, Ethiopia; 18 Department of Global Health and Amsterdam Institute for Global Health and Development, Amsterdam University Medical Centers, AHTC, Tower C4, Paasheuvelweg 25, 1105 BP Amsterdam, The Netherlands; 19 Netherlands Pharmacovigilance Centre Lareb, ‘s-Hertogenbosch, the Netherlands

**Keywords:** Pharmacovigilance, Tuberculosis, Health system assessment

## Abstract

**Background:**

New medicines have become available for the treatment of drug-resistant tuberculosis (DR-TB) and are introduced in sub-Saharan Africa (SSA) by the national TB programs (NTPs) through special access schemes. Pharmacovigilance is typically the task of national medicines regulatory agencies (NMRAs), but the active drug safety monitoring and management (aDSM) recommended for the new TB medicines and regimens was introduced through the NTPs.

We assessed the strengths and challenges of pharmacovigilance systems in Eswatini, Ethiopia, Nigeria and Tanzania, focusing on their capacity to monitor safety of medicines registered and not registered by the NMRAs for the treatment of DR-TB.

**Methods:**

Assessment visits were conducted to all four countries by a multidisciplinary team. We used a pharmacovigilance indicator tool derived from existing tools, interviewed key stakeholders, and visited health facilities where DR-TB patients were treated with new medicines. Assessment results were verified with the local NMRAs and NTPs.

**Results:**

Most countries have enabling laws, regulations and guidelines for the conduct of pharmacovigilance by the NMRAs. The relative success of NTP-NMRA collaboration is much influenced by interpersonal relationships between staff. Division of roles and responsibilities is not always clear and leads to duplication and unfulfilled tasks (e.g. causality assessment). The introduction of aDSM has increased awareness among DR-TB healthcare providers.

**Conclusion:**

aDSM has created awareness about the importance of pharmacovigilance among NTPs. In the future, a push for conducting pharmacovigilance through public health programs seems useful, but this needs to coincide with increased collaboration with between public health programs and NMRAs with clear formulation of roles and responsibilities.

**Supplementary Information:**

The online version contains supplementary material available at 10.1186/s12913-021-07043-6.

## Background

Pharmacovigilance is key to better understand the safety of medicines with the aim to prevent or minimize adverse drug reactions (ADRs), a major cause of morbidity and mortality [1–3]. In sub-Saharan Africa (SSA), 6.3% of hospital admissions were a direct consequence of ADRs, underscoring the need for well-functioning pharmacovigilance systems in SSA countries [3].

In SSA, public health programs (PHPs) play a key role in providing treatment for poverty-related infectious diseases including HIV/AIDS, malaria and tuberculosis (TB) [4]. National Tuberculosis Programs (NTPs) have a major role in the end-to-end care for TB patients, including diagnosis, treatment provision, and patient management and monitoring, usually in TB-specialized health facilities or TB units within general health facilities. Collaboration between National Medicines Regulatory Authorities (NMRAs) and PHPs can be delicate. In NTPs there has been ample attention for the management of ADRs in individual patients caused by anti-TB medicines, but until recently there was no attention to the importance of reporting ADRs to the NMRA to improve medicine safety on a population level [5, 6]. However, until shorter treatments with new medicines became available, ADRs did occur regularly in the then recommended 18- to 24-months, multiple-medicine regimens used for the treatment of multi-drug resistant (MDR) TB, [7] including hearing loss caused by aminoglycosides [8]. The need for adequate pharmacovigilance has become pressing with the introduction of new and re-purposed medicines for the treatment of (M)DR-TB which, because of the high clinical need, received accelerated or conditional regulatory approval from stringent regulatory authorities based on limited trial data [9–11].

Bedaquiline, the backbone of new, shorter, all-oral regimens, received accelerated approval from the United States Food and Drug Administration (USFDA) and conditional approval from the European Medicines Agency (EMA), based on two phase IIb trials including a total of 313 patients [9]. Likewise, delamanid was conditionally approved by EMA and the Japanese and Korean regulatory authorities based on two trials including 307 patients [10]. Pretomanid as part of a three-medicine, six-month, all-oral regimen was approved by USFDA for the treatment for specific forms of MDR-TB based on a study including 109 patients [11]. Re-purposed medicines, such as linezolid and clofazimine have never received regulatory approval for TB treatment and are being prescribed for longer durations than their labelled use, [6] and are used in new multi-drug regimens which may result in previously unknown drug-drug interactions [12].

These new medicines and regimens are being introduced globally through NTPs with the help of the Global Drug Facility (GDF) providing for quality assured medicines. In 2018, bedaquiline treatment was provided via a donation programme by the manufacturer and implemented by the United States Agency for International Development (USAID). Though in many countries not yet approved by the NMRA, these medicines are being used by thousands of difficult-to-treat TB patients, often under programmatic conditions, with limited resources for close patient monitoring [3]. This lack of safety monitoring can pose a serious risk to public health as countries cannot rely on safety data produced from the populations in which the medicines were developed [13]. Therefore, the World Health Organization (WHO) is recommending active pharmacovigilance for all patients on these new medicines and regimens through active Drug Safety Management and Monitoring (aDSM) [14]. A special database has been developed for the global collection of aDSM data by NTPs (see https://www.who.int/tdr/research/tb_hiv/adsm/en/), as an addition to the global pharmacovigilance database exploited by the Uppsala Monitoring Center on behalf of the WHO Program for International Drug Monitoring, which receives data on adverse drug reactions from NMRAs.

In the past, evaluation of pharmacovigilance systems has mainly focused on the role of the NMRAs, without taking into account the perspective of the PHPs, nor healthcare professionals (HCPs) perspectives [3, 15]. However, a pharmacovigilance system only works if there is collaboration between the NMRAs, PHPs and HCPs, especially in countries where PHPs play an important role in supplying medicines to patients [4, 5, 7].

The Pharmacovigilance Africa (PAVIA) project aims to strengthen pharmacovigilance systems in four SSA countries by fortifying the collaboration between PHPs and NMRAs, starting with the relationship between the NTPs and the NMRAs. To be able to measure improvements during the course of the project, a baseline assessment was done. An end-line assessment is planned to take place at the end of this 4-year project.

In this paper, we aim to update and deepen the insights on the strengths and challenges of pharmacovigilance systems in four SSA countries obtained from a previous assessment [3], with a focus on the capacity of the countries’ pharmacovigilance systems for monitoring safety of registered and non-registered medicines used in NTPs for the treatment of (M)DR-TB in order to minimize the occurrence of adverse drug reactions.

## Methods

### Aim of the assessment

To get a good understanding of the pharmacovigilance situation in relation to TB treatment at the start of the PAVIA project, we conducted a baseline assessment in all four countries. This assessment aimed to assess the readiness of the national systems for monitoring the safety or new medicines and regimens for the treatment of (M)DR-TB, and the ability of the system to effectively prevent occurrence of (preventable) adverse drug reactions.

### Design

The baseline assessment used a combination of structured group interviews using pre-structured assessment tools, in-depth interviews, and on-site observation. The assessment was conducted by a team of national and international experts in pharmacovigilance and MDR-TB during a one-week visit. Information collected during interviews was triangulated against documentation where available. Each assessment visit concluded with a half-day meeting in which findings were presented, discussed and endorsed by in-country key stakeholders. A full report was written for the NMRA of each country.

### Settings

The four countries being part of PAVIA were purposively selected because new medicines and regimens for MDR-TB were introduced and they represented different classes of pharmacovigilance system maturity, [3]: group 1 countries having minimal or no capacity for pharmacovigilance, group 2 countries having basic structures in place, group 3 countries having legal and organizational structures enabling them to collect and evaluate safety data, and group 4 countries having a pharmacovigilance system that detects, evaluates, and prevents medicine safety issues [3]. Selected for the project were the Kingdom of Eswatini (group 1), Ethiopia (group 2), Nigeria (group 4), and Tanzania (group 3). Information about the TB burden and the MDR-TB treatment program of the NTP in these countries provided in Table 1.

**Table 1:** Key indicators for (MDR) TB incidence and the MDR-TB treatment program of the NTP in four Sub-Saharan African countries

Country	TB incidence per 100,000 population (2017) [16]	MDR-TB incidence per 100,000 population (2017) [16]	Number of MDR-TB treatment centers (2018)	Number of MDR-TB patients (period) [16]	Number of MDR-TB patients receiving NDR (period)
**Eswatini**	308	31	13	702 (2018)	276 (2018)
**Ethiopia**	164	5.2	50	716 (2018)	110 (2018)
**Nigeria**	219	12	28	1786 (2017) [16]	33 (2017)
**Tanzania**	269	2.9	75	145 (2018)	129 (Nov 2017-Jul 2018)

The MDR-TB treatment centers usually initiate DR-TB treatment; once patients are stable on treatment, they are referred to satellite centers in all countries (except for Eswatini) to complete their treatment. In Ethiopia and Tanzania, patients have to visit the satellite center daily to receive treatment, while community-based treatment occurs in Eswatini and Nigeria. During their treatment, monthly checkup visits are scheduled at a DR-TB treatment center (Eswatini, Ethiopia, and Tanzania) or in a satellite center (Nigeria) in which specimens are collected to assess treatment response and other laboratory tests may be performed to check for adverse events

### Data collection

A pre-structured baseline assessment tool with pharmacovigilance indicators was developed including indicators from the WHO pharmacovigilance indicators list, [15] the Indicator-based Pharmacovigilance Assessment Tool, [17] and indicators developed by East African Community member states. The indicators addressed health system, policies, laws and financing, pharmacovigilance processes, capacity and infrastructure including training needs, stakeholder environment and communication/dissemination opportunities. A separate tool was developed for the NTPs, including indicators such as the total number of patients on MDR-TB treatment, on new medicines and regimens, and included in active pharmacovigilance schemes. The tool also assessed the collaboration with the national pharmacovigilance authorities, flow of ADR reports, and roles and responsibilities for assessing the ADRs including causality assessment. The tool is available in Supplementary File 1.

Short interview guides with key questions and discussion points were prepared (see Supplementary File 2). In-depth information was collected through face-to-face interviews and guided group interviews with the stakeholders and by observation during site visits to healthcare facilities treating patients with new medicines and regimens for MDR-TB. During interviews with stakeholders, the baseline assessment tools were filled, and information was triangulated against documentation where available. Additional information was captured in minutes for each in-depth interview and stored in Word files. For HCPs, questions focused on the providers’ knowledge about adverse events, their treatment and reporting to national authorities, as well as the providers’ experience reporting such events, including enablers and barriers for reporting. Data for the pharmacovigilance- and NTP assessment tool were collated by indicator in Excel files.

Quantitative data was obtained as much as possible before the in-country visit by a local team. Legal documents (decrees, policies, guidelines and essential medicines lists) concerning pharmacovigilance, bulletins and databases, annual reports and strategic plans of the NMRA and NTP, pharmacovigilance and aDSM guidelines and databases and standard operating procedures (SOPs) (where available) were used to extract data. If available, scientific papers from the countries assessed describing pharmacovigilance in general and in relation to (MDR) TB were also included.

### Data synthesis

For each country, all documentation shared with the assessors was reviewed before the in-country visit to get basic understanding of the laws and policies availing in the country. The in-country work started with a one-day kickoff meeting for the PAVIA project, followed by assessment visits to all main stakeholders: management of departments tasked with pharmaceutical policies and planning at the Ministry of Health, the national pharmacovigilance center (management and staff were interviewed and observations were done), NTP (focal persons for pharmacovigilance/aDSM and MDR-TB), and MAHs (4–8 different large companies per country, except in Eswatini, where there are no MAHs). Additionally, in Ethiopia and Tanzania, also universities and other public health programs, and in Eswatini and Nigeria, technical agencies partnering on pharmacovigilance were included. A representative set of 4–6 health facilities treating MDR-TB patients with new medicines and regimens were visited, including the MDR-TB clinic and the pharmacy. Interviews were held with clinic management, MDR-TB clinicians and nurses, and pharmacists. In Nigeria, the security situation did not permit clinic visits; instead, representatives of two hospitals (a pharmacist and a clinician) were interviewed in Abudja. The assessors were split over different teams, consisting of at least one international and several national assessors with backgrounds in pharmacovigilance or TB. At the end of every day, the teams shared results collected during the day and discussed emerging themes from these, solved unclarities and identified gaps to be addressed in the remaining in-country visit days. Together, the teams ensured that the assessment tools were filled and that additional clarification and specification was obtained through interviews. The information was summarized per theme. The main findings were presented during a half-day closure meeting with all key stakeholders to check if they matched the real situation. After the visit, remaining (quantitative) data gaps were filled and a final baseline assessment report including recommendations was written. The country reports were reviewed and approved by the country’s primary stakeholder in this process: the NMRA.

As most of the data collected are of qualitative nature, no statistical analyses were conducted.

## Results

### Policy, law and regulations

Table 2 presents an overview of the available acts, proclamations, regulations, and policies in the four countries.

**Table 2:** Legal provisions for pharmacovigilance in four Sub-Saharan African countries, 2018

Country	Date of assessment	Policy, year of reinforcement(year of last update)			Proclamation/ Act		Regulations		Guidelines	
		Nationalhealthpolicy	Nationaldrugpolicy	StandalonePV policy	title and/ ornumber, year	legal provisionsfor PV?	title and/ ornumber, year	legal provisionsfor PV?	General PV guidelines	For aDSMspecifically
**Eswatini**	25–29 Jun 2018	2006 (2011)	2011 (NA)^b^	NA^a^	Medicines & RelatedSubstances ControlAct (Act no. 9), 2016	No	NA	NA	NA	NA^c^
**Ethiopia**	27–31 Aug 2018	1993 (2000)^a^	1993 (2000)^d^in Health Policy	NA	ProclamationNo. 661/2009	Yes	No. 299/2013^a^, 2013	Yes	ADR Guideline, 2018Guideline for AdverseDrug EventsMonitoring[Pharmacovigilance],2018	NA^c^
**Nigeria**	24–28 Sept 2018	2004 (NA)	2003 (2005)	2012^a^	Decree 15, 1993 Nowreferred to as ActCap N1 Laws of theFederal Republic ofNigeria, 2004	Yes	National PVimplementationframework	No	NAFDAC GoodPharmacovigilancePractice Guidelines,2016	2018
**Tanzania**	30 Jul-5 Aug 2018	2007 (NA)	1991 (2007)	2018	Medicines andMedical Devices Act,Cap 219, 2003	Yes	Pharmacovigilanceregulations, 2018	Yes	National Guidelines forMonitoring MedicinesSafety, 2018	NA^c^

^a^(update) expected in 2019/2020

^b^In Eswatini, there is a National Pharmacy Policy, not a National drug policy

^c^For Eswatini, there is a very short section about aDSM in the national guidelines “Bedaquiline and Delamanid for the Treatment of Drug Resistant Tuberculosis: Guidelines for Clinicians” (SIAPS, 2015), for Ethiopia, a section is included in the National Guideline for drug-resistant tuberculosis (NTP, 2018, 2nd edition), for Tanzania, there is a training manual on aDSM, which does put focus on the management of aDSM rather than the reporting of ADR

^d^included in National Health Policy

All countries had a national health policy in place. A national drug policy was available in three of the four countries, but as part of the health policy in Ethiopia. Eswatini had a national pharmacy policy, but not a drug policy. A standalone pharmacovigilance policy was enforced in Nigeria and Tanzania. Acts providing for the establishment of the NMRA were in place in all four countries. However, in Eswatini, although the Medicines and Related Substances Control Act of 2016 provided for the establishment of an NMRA within one year after enforcement, this was not achieved due to funding constraints. Instead, a Medicines Regulatory Unit (MRU) implemented some of the functions of the NMRA, e.g. pharmacovigilance. Also, in the Act of Eswatini, there are no legal provisions for pharmacovigilance or medicine safety. Because of this, no general pharmacovigilance guidelines are available in the country. Probably related to this, pharmacovigilance had no clear place in national policy documents, including national strategic plans of PHPs.

While all regulatory authorities had pharmacovigilance embedded in their tasks, only in Tanzania, the directorate responsible for pharmacovigilance was directly funded by the Government. In the other countries, the NMRA’s budget was rather internally distributed over its (sub) directorates according to the agency’s prioritization.

In 2018, most countries were yet to have a guideline and SOPs for the conduct of aDSM (Table 2). Only the Nigerian NTP had a guideline for aDSM, developed in collaboration with NAFDAC and other partners, but no SOP for filling in the aDSM form. In Ethiopia and Tanzania, National Technical Working Groups developed an implementation plan for the new drugs and regimens which included a section on pharmacovigilance/aDSM. While in Ethiopia, the aDSM guideline had been developed jointly by the NTP and the NMRA from the start, in Tanzania, the Tanzania Medicines and Medical Devices Authority (TMDA) got involved after the first draft had been developed.

The Eswatini NTP had developed a training manual on the use of new medicines with a special focus on the identification and management of ADRs. Similarly, the Tanzanian NTP had developed a training manual on aDSM with focus on the management rather than the reporting of ADR, while an SOP for completion of the aDSM form was in preparation.

### Systems, structure and stakeholder coordination

The stakeholder landscape for NTPs is complex and involves multiple stakeholders with different interests, which, for most, is not focused on pharmacovigilance. NMRAs were historically not regarded as key stakeholders by NTPs but this is currently changing, triggered by the requirement of “active pharmacovigilance” (later specified as aDSM) by the global TB program at WHO. In its updated national strategic plan of 2018, the NTP of Ethiopia mentioned EFDA in supporting TB activities related to medicinal product registration, regulations, medicine quality, and aDSM [18]. In Nigeria, NAFDAC was actively involved in developing the aDSM guideline and reporting forms as well as a training for HCPs. In Eswatini, driven by visionary focal persons maintaining strong personal relationships, a close and effective collaboration was set up between the NTP and the pharmacovigilance unit, which fostered the joint development of tools and trainings for the monitoring of adverse events for the new MDR-TB medicines. In contrast, in Tanzania, the TMDA was not involved in aDSM activities.

Natural stakeholders of the NTPs are HCPs in TB hospitals, clinics and community health centres, and related staff. Quarterly meetings between focal persons for the DR-TB program and clinical DR-TB experts are common. In such meetings, ADRs can be discussed as a part of patient management; sometimes, advocacy activities on pharmacovigilance are included (e.g. in Eswatini), but the NMRAs are not always represented (e.g. Eswatini, Tanzania). In other countries however (Ethiopia, Nigeria), most activities are done jointly. For example, in Ethiopia, NTP and EFDA jointly conduct pharmacovigilance trainings and supervision visits, and there is a National Clinical Review Committee for DR-TB in which EFDA is also represented.

While different donors provided the necessary precondition of funding, several technical partners have assisted in strengthening pharmacovigilance programs or in the conduct of specific pharmacovigilance activities. For example, the Strengthening Pharmaceutical Systems project funded by USAID made an important contribution to pharmacovigilance strengthening in Eswatini. In the same country, Médicins sans Frontiers conducted active surveillance on the effectiveness and safety of a simplified short regimen for MDR-TB treatment. In Nigeria, IHVN was providing support to development of aDSM forms, and KNCV helped with the development of aDSM guidelines. KNCV was also assisting in development of aDSM guidelines, reporting forms and SOPs in Ethiopia and Tanzania.

### Reporting flow

Table 3 summarizes the reporting forms available, who was responsible for receiving these, and how the adverse event information was shared between stakeholders.

**Table 3:** ADR reporting for adverse events in patients on anti-tuberculosis treatment^a^

Country	Form(s) used for reporting:			Authority to which primary ADR report is submitted	aDSM expert committee	Reporting to PIDM^a^ database	Reporting to global aDSM database^**b**^	Reporting to GDF^c^
	TB- Specific form	Yellow card form	GDF Form					
Eswatini	X^d^	X		NMRA	NA^e^	MoH (NPVU)	NTP	not done
Ethiopia	X^f^	X	X^g^	NMRA	NA^h^	EFDA	not done	not done
Nigeria	X	X	X^g^	NTP	X	NAFDAC	not done	NTP
Tanzania	X^i^	X^i^	X^g^	NTP and NMRA^i^	X	TMDA	not done	TMDA

^a^Abbreviations: *ADR* adverse drug reaction, *aDSM* active drug safety monitoring and management, *EFDA* Ethiopian Food and Drug Administration, *GDF* Global Drug Facility, *MoH* Ministry of Health, *NA* not available, *NAFDAC* National Agency for Food and Drug Administration and Control, *NMRA* national medicines regulatory authority, *NPVU* national pharmacovigilance unit, *NTP* national tuberculosis program, *PIDM* Programme for International Drug Monitoring, *PV* pharmacovigilance, *TMDA* Tanzania Medicine and Medical Devices Authority, *TB* tuberculosis

^b^ WHO and TDR (Special Programme for Research and Training in Tropical Diseases; https://www.who.int/tdr/news/2016/global-database-adsm/en/) host the global aDSM database.

^c^ Countries receiving bedaquiline under the USAID donation programme had to send reports of serious adverse events to the GDF (www.stoptb.org/gdf/)

^d^At the time of assessment (June 2018), different forms were used: a TB-specific form in sentinel sites, a project-specific form for TB patients used in one site, and Yellow card forms for aDSM. After harmonization, there are now two data collection forms, one for spontaneous reporting on any adverse event, and one for active pharmacovigilance (aDSM data for MDR-TB and dolutegravir containing regimens from selected facilities)

^e^A National Patient safety Management and Monitoring committee assesses all types of ADRs from all types of medicines.

^f^Adverse events of special interest can be entered into an Excel sheet by the TIC’s DR-TB clinicians or nurses (called *line listing* in the system). This sheet is monthly sent by email to EFDA (with a copy to the focal person of NTP). Serious adverse events are reported through the Yellow card form

^g^ Filled and submitted by the NTP.

^h^There is a clinical review committee at the NTP and a safety advisory committee at EFDA

^i^TB related ADRs should be reported both using the aDSM form and the Yellow Card form

There were several means of reporting available, some of which were designed especially for aDSM. In Nigeria and Tanzania, the NTP was the first recipient of the aDSM report. Reports were thereafter shared with the pharmacovigilance centre in the NMRA. In both countries, spontaneous reports were sent straight to the pharmacovigilance centre using the yellow card reporting form. In Eswatini, the pharmacovigilance unit was the primary recipient of ADR reports and shared them with the NTP. In Ethiopia, EFDA received both yellow card reporting forms (for spontaneous reporting) and a line listing with information on adverse events collected in the scope of aDSM.

Though reporting of serious adverse events with the new medicines to the GDF was put as a requirement to NTPs receiving bedaquiline under the USAID donation program, not all NTPs reported to the GDF. Forwarding the reports to the WHO Program for International Drug Monitoring (PIDM), maintained by the Uppsala Monitoring Centre, was in all countries the task of the pharmacovigilance centre within the NMRA. Only the NTP of Eswatini also reported its ADRs to the global aDSM database (Table 3). The existence of different international databases (PIDM, aDSM and GDF) is expected to have caused partially duplicate reporting.

The aDSM expert committee, discussing aDSM reports, in Nigeria and Tanzania fell under the responsibility of the NTP. Eswatini and Ethiopia did not have an aDSM committee. In Ethiopia, a clinical review committee under supervision by the NTP, discussed the clinical management of complex MDR-TB cases and also provided causality assessment for reports of serious adverse events from MDR-TB patients.

### Risk assessment and evaluation

#### Marketing authorization holders (MAHs)

Usually MAHs are responsible for monitoring the safety of their products including risk assessment and evaluation. They are obliged to send periodic safety update reports to the NMRA evaluating the risk-benefit balance of the products they sell, provide for risk management and pharmacovigilance plans.

However, in 2018, new medicines for DR-TB treatment were not all registered by the NMRAs and hence, no MAHs were involved in making these medicines available to patients in the countries. Instead, these medicines entered the countries under strategic (or accelerated) access schemes, which are special arrangements between the regulators and the NTPs, usually meant for testing medicines or other supplies for (operational) research purposes. For non-registered medicines, pharmacovigilance is not legally mandatory, this mechanism thus bypasses the legal system as no MAH takes mandatory responsibility for reporting of ADRs.

#### National medicines regulatory agencies (NMRAs)

All four countries had spontaneous reporting systems in place, although these received few reports (Table 4).

**Table 4:** ADR reporting rates in 2017^a, b^

Country	Numberof reportsreceivedat the PVcentre	Millioninhabitants	Reportsper millioninhabitants	Number ofTB reportsreceived bythe NTP	Numberofpatientswith TB	Reports per1000 TBpatients	Number ofreports frompatientsincluded inaDSM	Numberofpatientson NDR	aDSM reportsper 1000patientson NDR
Eswatini	224^c^	1.2	187	NA (9 MDR-TB)	1374(318 MDR-TB)	NA (28.3 MDR-TB)	19^d^	276^d^	69
Ethiopia	706	105	6.7	67	117,705	0.6	28^e^	218^e^	128
Nigeria	2173	186	11.7	1210 (156)^f^	109,637	11.0 (1.4)^f^	5	33	152
Tanzania	287	60	4.8	49	69,818	0.7	114^g^	129^g^	884

^a^ Unless otherwise indicated

^b^Abbreviations used in this table: *ADR* adverse drug reaction, *aDSM* active drug safety monitoring and management, *NDR* new drugs and regimens (i.e. bedaquiline, delamanid, and a shorter treatment regimen), *PV* pharmacovigilance, *TB* tuberculosis

^c^June 2016 – May 2017

^d^January 2016 – December 2018

^e^July 2016 – June 2018

^f^Of the 1210 reports received by the NTP, only 156 were forwarded to NAFDAC. The number of reports per 1000 TB patients was calculated both for all reports received by the NTP, and for the reports that reached the NMRA (in brackets)

^g^November 2017 – July 2018

This does not mean there is no attention to ADRs in clinical practice. In the TB treatment facilities, there was awareness among clinicians, nurses and laboratory staff about the occurrence and management of adverse events, and patients were actively questioned and investigated for adverse events. For example, in Eswatini, while a checklist with the most commonly occurring adverse events is used during the monthly check-up visit, nurses are trained to ask open questions about adverse events. Also, community-based treatment observers receive training in recognizing adverse events and can report these to treatment facility by telephone. Ancillary medicines were available free of charge, be it with varying availability across the clinics visited. Despite this awareness, the implementation of aDSM has been difficult in some places where equipment for monitoring such as ECG and audiometry has been lacking, been faulty or where staff were unable to operate it.

HCPs had no routine of documenting and transmitting suspected ADRs to the pharmacovigilance centre. This was partly due to limited awareness about the importance of reporting, the types of adverse events to be reported, and work overload. Reporting was generally seen as a burden as it is time consuming, pharmacovigilance activities were not mentioned in the healthcare workers’ job description, and there were no direct incentives to report adverse events. Also, although acknowledgement of receipt was usually sent, individual feedback on reports was not provided. Table 5 describes how reports submitted to the NMRA were processed.

**Table 5:** Handling of ADR reports received by the NMRA^a^

Country	Acknowledgementof receipt report	Individualfeedbackreport	Aggregatedfeedback reports	Causality assessmentand signal detection	Joint causalityassessmentNMRA/NTP
Eswatini	Not consistently	No	Yes	No	No
Ethiopia	Yes	No	Yes	Yes^b^	Yes^d^
Nigeria	Yes	No	Yes	Yes	No
Tanzania	Yes	No	Yes	Yes	Yes^c^

^a^Abbreviations used in this table: *ADR* adverse drug reaction, *NMRA* national medicines regulatory authority, *NTP*, national tuberculosis program

^b^Only 10 reports were subjected to a formal causality assessment during the past calendar year, based on an assessment of case severity, and community/public health programme concerns. Most of these concerned AEFIs and adverse events among TB patients on new medicines

^c^Causality assessment is currently done by the National aDSM committee during quarterly meetings. Recently, it was agreed between NTP and TMDA that TMDA will take the lead in causality assessment and be represented in all National aDSM Committee meetings. NTP is responsible for organizing the meetings

^d^Causality assessment for adverse event reports from DR-TB patients through the aDSM scheme is done during clinical review meetings. Those adverse events that have fatal outcomes are prioritized. The National Clinical Review Committee consists of staff members from the Medical Faculty of Addis Ababa University with different specializations as well as DR-TB specialized clinicians from the two DR-TB centres of excellence (St Peter’s and ALERT hospital), representatives of the NTP and partner organisations, and a representative of EFDA. NTP is responsible for organizing these meetings. Whenever needed, email consultations are organized, but the Committee sometimes also physically meets. If needed, the Clinical Review Committee can visit the TIC to verify the information in the ADR report and collect additional information

ADR reports were entered into central databases in all countries. Most countries used Excel for this, for example, because the reports received were often incomplete prohibiting submission to VigiFlow. Only in Tanzania, all ADR reports were directly entered in VigiFlow. In other countries, although it was the intention to enter all reports in VigiFlow, only a selection was entered, due to issues with incomplete reports (Nigeria) or work load (Ethiopia, Eswatini). Excel databases were not (Ethiopia, Eswatini) or not often (Nigeria) backed up, and there was no dedicated computer available for the storage of the central database in Eswatini, where the electronic storage of pharmacovigilance data was fully donor-driven. Reports received via the aDSM program were entered in a separate database in all countries except Eswatini. Only in Ethiopia, this database was located at the NMRA. It was not possible to (automatically) extract data from the aDSM database to the general pharmacovigilance database.

### Risk management and communication

Table 6 summarizes the number of signals communicated and detected between 2013 and 2017, and regulatory actions taken. Eswatini does not have a medicine regulatory authority and can therefore not take regulatory action. However, the MRU within the MoH acted on signals. None of these actions concerned TB medicines. In Nigeria, this information was always communicated through letters to HCPs, while in Ethiopia and Tanzania, the regulatory actions were primarily communicated though a medicine safety bulletin. However, this was not issued as often as intended, so that new information did not easily reach the HCPs and patients. In Eswatini the pharmacovigilance group within the MRU also used WhatsApp and e-mail for urgent safety messaging.

**Table 6:** Signals^a^ detected in four different sub-Saharan countries, 2013–2017

Country	# signals detected	# signals based on national data	# regulatory actions taken based on signals	# signals for TB medicines
Eswatini	2	0	0	0
Ethiopia	0	0	31^b^	0
Nigeria	0	0	0	0
Tanzania	1	1	10	0

^a^For a definition of this term, see https://www.ema.europa.eu/en/documents/scientific-guideline/guideline-good-pharmacovigilance-practices-gvp-module-ix-signal-management-rev-1_en.pdf

^b^These regulatory actions were taken for the general medicine report, but were not based on signals detected

None of the NTPs had identified any safety signals in the past 5 years. For communicating TB related information, including information regarding the safety of TB medicines, different mechanisms were used, such as e-mail (Ethiopia) or WhatsApp (Nigeria), newsletters (Ethiopia and Tanzania), face-to-face meetings with healthcare providers (Nigeria), or a website (Eswatini).

## Discussion

We performed a baseline assessment of the pharmacovigilance situation in four SSA countries focusing on pharmacovigilance for anti-TB medicines. In 2018, three of the four countries had enabling laws, regulations and policies in place to perform pharmacovigilance. Only Tanzania had dedicated government funding for pharmacovigilance. Most NTPs had developed or updated forms and reporting structures for the reporting of adverse events, or were in the process of doing so. To further enhance pharmacovigilance in the TB programs in all four countries, the aDSM implementation framework was introduced, [14] mostly in collaboration with the NMRA.

There used to be little cooperation between the NTPs and the NMRAs and little priority given to pharmacovigilance in PHPs [5, 19]. This limits the national pharmacovigilance systems’ ability to prevent adverse drug reactions effecively. aDSM has increased the awareness about pharmacovigilance among the NTPs and the TB HCPs and has stimulated the collaboration with the NMRAs, particularly in Ethiopia and Eswatini. In these countries, the NTP and NMRA together have managed to set up an efficient reporting system in which all reports are directly sent to the NMRA and from there, after assessment and entry, are shared with the NTP.

Enabling legal provisions are important for setting up a strong pharmacovigilance program [20]. Of the four countries assessed, Nigeria had the strongest legal system including a PV policy, guidelines and SOPs. However, even though there is a legal basis, the success of collaboration between PHPs and NMRAs is dependent on personal relationships. With a strong dedicated officer for pharmacovigilance in the NTP, and mutual respect of NTPs and NMRAs organization’s vision, needs and wishes, successful systems can be developed. With sufficient funding and staffing on both sides, such bonds can be built [21]. Still, good relationships are needed to boost collaboration. Examples are Eswatini, where much of the legislation is not yet in place but strong visions and personal relationships have driven implementation of the aDSM system, and Ethiopia, where EFDA takes a central role in collecting pharmacovigilance reports including aDSM reports and submitting these to the WHO global database**,** and jointly conducts trainings and supportive supervision with the NTP. 

aDSM was pressed for by the WHO TB program demanding “active pharmacovigilance” for the use of bedaquiline, [22] and delamanid, [23] which was followed by assistance to NTPs from funders and technical agencies in setting up aDSM in TB health facilities [7]. However, as this pressure has come through TB channels and guidance was provided to NTPs only, this has also led to duplication of systems and reporting forms – even though the aDSM framework document recommends collaboration with national pharmacovigilance authorities to avoid the creation parallel systems [14]. However, the creation of a specific international aDSM database contradicted rather than underlined the importance of this recommendation.

We observed that in some countries (e.g. Tanzania), TB HCPs were supposed to report in duplicate for each adverse event. Also, some countries (e.g. Eswatini) are reporting to several international pharmacovigilance databases, which will lead to partially duplicate information gathering in different hubs, and scattered, incomplete safety profiles. In situations where staff is scarce, it will take away manpower from other pharmacovigilance tasks. Duplication of efforts was one of the barriers to reporting of adverse events mentioned by HCPs in a qualitative study [24].

Time constraints, lack of priority to the reporting of adverse events and not receiving individual feedback on their reports or other types of incentives, were also mentioned by the HCPs we interviewed. HCPs focus on the treatment of their patients (including the management of adverse events), and do not necessarily see that information from individual patients can benefit the treatment and safety of other patients through the reporting of adverse events [25]. Also time constraints, [24] fear of legal liability, [25] and lack of knowledge about pharmacovigilance, [25–27] are barriers for ADR reporting. While the introduction of aDSM has helped in addressing the knowledge and awareness gap, it did not solve issues of time constraints and duplication of efforts [7].

From a legal point of view, the NMRA is responsible for monitoring the safety of the medicines which they have approved for marketing in the country. However, the new medicines and regimens for TB treatment were not yet available on the market at the time of introduction in the NTP. Instead, these medicines were imported under special (accelerated) access schemes, directly by the NTPs. For other medicines, the NTPs are only responsible for their procurement and distribution, not for pharmacovigilance, and they have no or inadequate legal mandate and guidelines for executing this. Thus, the roles and responsibilities for the safety monitoring of these new medicines remain unclear. We have observed that this sometimes led to initial tension (e.g. Nigeria), unclarities about who is responsible for specific tasks, such as causality assessment (e.g., Eswatini), entering aDSM reports into databases (e.g. Ethiopia), and duplication of work (e.g. Nigeria, Tanzania).

NMRAs are looking for mechanisms to ease reporting by HCPs. An example is the use of WhatsApp in Eswatini. It should be investigated if any privacy issues arise; otherwise other mobile reporting systems may be preferred, such as the Med Safety App (https://web-radr.eu/mobile-apps/med-safety/).

### Limitations

Our analysis is limited in several ways. First, this assessment followed the routine assessments as recommended by WHO, [15] using existing assessment tools [15, 17]. No established qualitative research methods were used [28]. This may have limited collection of data outside the assessment tools. Therefore we added in-depth interviews with key stakeholders using a set of key discussion points and questions, and probed interviewees outside these points if what they shared seemed relevant to describe the country’s pharmacovigilance situation. Second, we only could include the view of those interviewed and do not know if their views and experiences are generalizable for the whole country. Interviewees, including HCPs, were often located in the capital and we thus may not have sufficiently captured practices in rural and remote areas. To ensure maximum generalizability, we tried to include a wide range of stakeholders and site visits to health facilities outside the capital. This assessment only included a one-week visit with a limited number of assessors. Therefore, certain relevant aspects, such as financial aspects, the functioning of electronic databases and their contents, and patient views could not be included.

## Conclusions

In conclusion, aDSM has created awareness about the importance of pharmacovigilance among NTPs. Collaboration between PHPs and NMRAs can be delicate especially when new medicines and regimens enter the countries through special access schemes. In some countries, it remains unclear who bears responsibility for medicine safety monitoring in these situations.

The confusion about roles and responsibilities for the pharmacovigilance of new (not registered) medicines introduced through PHPs can be avoided by providing for legal guidance. Also legal provisions for sharing information regarding ADRs between PHPs and NMRAs are needed.

Duplication of efforts can be prevented by introducing a robust electronic system that can be accessed by all partners involved in pharmacovigilance. Ideally, each partner would have its own reading and editing rights, so that different partners can fill and learn from one and the same system. If one system is not feasible, then smooth transfer of information between systems should be possible [29].

In the future, a push of conducting pharmacovigilance in PHPs seems useful, but this needs to coincide with clear formulation of roles and responsibilities of both the PHPs and the NMRAs.

## Supplementary Information

**Additional file 1.**

**Additional file 2.**

## Data Availability

All quantitative data collected in the assessment are shared in this manuscript. The full assessment reports can be requested by contacting the country’s NRMA via Siphesihle Nhlabatsi (NPVU Eswatini, ntinisphe@gmail.com), Asnakech Alemu (EFDA Ethiopia, aalemu@efda.gov.eth), Abiola Sadikat Abiodun (NAFDAC Nigeria, abiola.abiodun@nafdac.gov.ng), and Kissa Mwamwitwa (TMDA Tanzania, ki313ssa@yahoo.com).

## Abbreviations

ADR: Adverse drug reaction
aDSM: Active drug safety monitoring and management
DOT: Directly observed treatment
DR: Drug resistant
EFDA: Ethiopian Food and Drug Administration
EMA: European Medicines Agency
GDF: Global Drug Facility
HCP: Healthcare professionals
IHVN: Institute of Human Virology Nigeria
MAH: Marketing authorization holder
MDR: Multidrug resistant
MRU: Medicines Regulatory Unit
NMRA: National medicines regulatory agency
NAFDAC: National Agency for Food and Drug Administration and Control
NPVU: National pharmacovigilance unit
NTP: National Tuberculosis Programme
OPD: Outpatient department
PAVIA: Pharmacovigilance Africa
PIDM: Program for International Drug Monitoring
PHP: Public Health Programmes
PMDT: Programmatic Management of DR-TB
SOP: Standard operating procedure
SSA: Sub-Saharan Africa
TB: Tuberculosis
TMDA: Tanzania Medicines and Medical Devices Authority
TIC: Treatment Initiation Center
USAID: United States Agency for International Development
USFDA: United States Food and Drug Administration
WHO: World Health Organization
