# Supplementary material for: Baseline assessment of pharmacovigilance activities in four sub-Saharan African countries: a perspective on tuberculosis
Source: BMC Health Serv Res. 2021 Oct 8;21:1062. doi: 10.1186/s12913-021-07043-6 (PMC8499544; doi:10.1186/s12913-021-07043-6)
Supplement: Supplementary file 1 — Additional file 1. [file 12913_2021_7043_MOESM1_ESM.pdf]

Indicator #	Indicator	Assessment Questions	Answer	Notes & additional comments	Information source
**Component 1. Policy, Law, and Regulation**					
1.1	Existence of a policy document that contains essential statements on pharmacovigilance or safety of medicines, health products and technologies (stand alone or as a part of some other policy document)	Is there a national policy on pharmacovigilance or medicine safety, or a more general medicines policy that contains essential statements? *Yes/No*<br>*Request documentation to verify.*			
		When was the policy last reviewed? *Date*<br>*Request documentation to verify.*			
1.2	Existence of specific legal provisions for pharmacovigilance in the national medicines legislation or similar legislation	Are there legal provisions for pharmacovigilance or medicine safety in the medicines act or law? *Yes/No*<br>*Request documentation to verify.*			
1.3	Legal provisions for Marketing Authorization Holders to monitor and report the safety and quality of their products	Is it mandatory by law or regulations for marketing authorization holders to conduct post marketing safety activities? *Yes/No*<br>*Request documentation to verify.*			
		Is it mandatory by law or regulations for marketing authorization holder to report adverse drug reactions/medicine safety related issues? *Yes/No*<br>*Request documentation to verify.*			
		Is it mandatory by law or regulations for marketing authorization holders to regularly submit periodic safety update reports (PSUR) or periodic benefit-risk evaluation reports (PBRER)? *Yes/No*			
		If yes, which are the required time intervals?			
1.4	Existence of legal provisions empowering the national regulatory authority to require Marketing Authorization Holders to submit proof of their proactive pharmacovigilance planning as part of an application for product licensing	Does the national regulatory authority have the power to require Marketing Authorization Holders to submit any of the following documents prior to product licensing?<br>I. Pharmacovigilance plan			
		II. Risk management plan			
		III. Risk minimization/mitigation plan			
		Are Marketing Authorization Holders required to adapt the plans to the particular risk situation of the population in the country?			
1.5	Existence of national pharmacovigilance guidelines developed or reviewed within the past 5 years	Does a national guideline for pharmacovigilance (or a related document) exist? *Yes/No*<br>*Request documentation to verify.*			
		Has the national pharmacovigilance guideline been developed or reviewed within the past 5 years? *Yes/No*<br>*Request documentation to verify.*			
		When were the guidelines last reviewed? *Date. Request documentation to verify.*			

Indicator #	Indicator	Assessment Questions	Answer	Notes & additional comments	Information source
1.6	Regulations and guidelines encourage distributors, importers exporters, health- care institutions, other stakeholders and consumers to report ADR and/or AE to the MAH and/or NRA	Do regulations and guidelines encourage distributors, importers exporters, health-care institutions, other stakeholders and consumers to report ADR and/or AE to the MAH and/or NRA, *Yes/No , Request documentation to verify.*			
1.7	The legal provisions and/or regulations allow NRA to require manufacturers and/or MAHs to conduct specific studies on safety and effectiveness under specific conditions	Does the national regulatory authority have the mandate to require manufacturers and/or marketing authorization holders to conduct and present results from specific studies addressing identified safety concerns? *Yes/No, Request documentation to verify.*			
1.8	Legal provisions, regulations and/or guidelines require manufacturers and/or marketing authorization holders to designate a Qualified Person responsible for vigilance.	Do legal provisions, regulations and/or guidelines require manufacturers and/or marketing authorization holders to designate a Qualified Person responsible for vigilance? *Yes/No, Request documentation to verify.*			
1.9	Existence of updated National Essential Medicines List that was reviewed with consideration of medicine safety information	Is there an essential medicines list in use? *Yes/No, Request documentation to verify.*			
		Does the essential medicines list selection committee consult medicine safety information? *Yes/No*			
		When was the list last reviewed? *Date, Request documentation to verify.*			
1.10	Existence of a medicines regulatory authority or agency	Is there a drug regulatory authority or agency? *Yes/No*			
1.11	Existence of official records of licensed medicinal products	Is there an official source of information on medicinal products that are licensed for use in the country? *Yes/No* *Request documentation to verify.*			
1.12	Accreditation of private health facilities includes requirements for the existence of a pharmacovigilance system	Does the public authority responsible for accreditation of private health facilities require that a pharmacovigilance system is in place? *Yes/No, Request documentation to verify.*			
**Component 2. Systems, Structures, and Stakeholder Coordination**					
2.1	Existence of a national pharmacovigilance center with a clear mandate and structure	Is there a National pharmacovigilance center or any other body assigned the responsibility of monitoring safety of medicines? *Yes/No*			
		Is there a clear mandate and organizational structure for the pharmacovigilance center? *Yes/No* *Request documentation to verify.*			

Indicator #	Indicator	Assessment Questions	Answer	Notes & additional comments	Information source
2.2	The pharmacovigilance center has designated, qualified human resources to carry-out its functions	How many staff members (full-time equivalent) does the PV center or system have who are specifically responsible for carrying out its functions (technical and administrative)? *Request documentation to verify.*			
2.3	Existence of a dedicated financial provision or statutory budget for the pharmacovigilance center	Is there an annual budgetary allocation for pharmacovigilance activities or for the Pharmacovigilance Center? *Yes/No* *Request documentation to verify.*			
		In the last fiscal year, how many funds were allocated by the government and donors for pharmacovigilance activities? *Please enter the amount in the Answer box and specify the currency in the Notes column. Request documentation to verify.*			
2.4	Existence of a functional national medicine safety advisory committee	Does a national medicine safety advisory committee exist with the responsibility to provide technical advice on the safety of medicines to the regulatory authority? *Yes/No,* Request documentation to verify.			
		Has the national medicine safety advisory committee met at least twice in the previous calendar year? *Yes/No* *Request documentation to verify.*			
2.5	Existence of standard operating procedures (SOPs) for conducting pharmacovigilance activities	Does the NMRA / PV center have SOPs for pharmacovigilance activities? *Yes/No, Request documentation to verify.*			
		When were the SOPs last reviewed? *Date. Request documentation to verify.*			
2.6	Existence of a source of data on consumption and prescription of medicines	Are there any sources of information on sales or consumption of medicines on a national, regional or local level? *Yes/No* *Request documentation to verify.*			
		Are they publicly available? *Yes/No Request documentation to verify.*			
2.7	Existence of a library or other reference source for drug safety information	Does the pharmacovigilance center has access to a library or electronic sources providing up-to-date information on medicine safety and the progress of scientific knowledge in the domain? *Yes/No Request documentation to verify.*			
2.8	Existence of a mechanism to disseminate pharmacovigilance information (including one or more of the following: newsletters, information bulletin, website or phone line for dissemination of pharmacovigilance information)	Is there a communication plan in place to disseminate PV information? *Yes/No, Request documentation to verify.*			
		Is there a newsletter or information bulletin for dissemination of PV information? *Request documentation to verify.*			
		- How many issues of the medicine safety bulletin are supposed to be published per year?			
		- How many issues of the medicine safety bulletin were published in the previous calendar year? *Request documentation to verify.*			
		Is there a website for dissemination of PV information?			
		Is there a publicly advertised phone line to receive and provide medicine safety and PV information? *Yes/No* *Request documentation to verify.*			

Indicator #	Indicator	Assessment Questions	Answer	Notes & additional comments	Information source
		Are findings published in national/international journals? *Yes/No, Request documentation to verify.*			
		Is there another mechanism for dissemination of PV information? *Please describe the mechanism in Notes*			
2.9	Existence of harmonized pharmacovigilance curricula for key healthcare workers - **Pre-Service***	Is PV incorporated into the national **pre-service** curricula of **doctors?** *Request documentation to verify.*			
		Is PV incorporated into the national **pre-service** curricula of **nurses?** *Request documentation to verify.*			
		Is PV incorporated into the national **pre-service** curricula of **pharmacists?** *Request documentation to verify.*			
2.10	Existence of harmonized pharmacovigilance curricula for key healthcare workers - **In-Service**	Is there a pharmacovigilance training module, manual, or curriculum for **in-service** training of health care workers? *Yes/No*<br>*Request documentation to verify.*			
2.11	Number of healthcare workers trained in pharmacovigilance in the previous calendar year through in-service training program	How many healthcare workers has the center/program trained on PV in the previous calendar year (through **in-service** training)? *Request documentation to verify.*<br>a. Health professionals			
		b. Community health workers			
		How many training events/sessions were conducted in the previous calendar year? *Request documentation to verify.*<br>a. For health professionals			
		b. For community health workers			
2.12	Adoption and use of harmonized web-based pharmacovigilance training tools	Are web-based pharmacovigilance training tools available?<br>a. For health professionals *Yes/No*			
		b. For the general public *Yes/No*			
2.13	Existence of a functioning platform, mechanism or strategy for the coordination of pharmacovigilance activities - **National Level**	Does a platform, mechanism or strategy for the coordination of pharmacovigilance activities (such as PV technical working group, forum or regularly scheduled meetings) exist among **national stakeholders**? *Yes/No Request documentation to verify.*			
		Have the key **national stakeholders** convened at least once in the previous calendar year? *Request documentation to verify.*			
2.14	Submission of AE reports by health-care facilities in the previous year	From how many health facilities were AE reports received in the previous calendar year?			
		How many health facilities are there in the country?			
		How many health facilities submitted >10 reports to the PV center in the previous calendar year?			
2.15	Evidence of consideration of safety data when developing and updating standard treatment guidelines	When developing standard treatment guidelines is evidence on safety data being described and taken into consideration? *Request documentation to verify.*			

Indicator #	Indicator	Assessment Questions	Answer	Notes & additional comments	Information source
2.16	National pharmacovigilance center is a full or associate member of the WHO Program for International Drug Monitoring	Is the national pharmacovigilance center a full or associate member of the WHO Program for International Drug Monitoring? *No member, associate member, full member.*			
**Component 3. Signal Generation and Data Management**					
3.1	Existence of a national database for pharmacovigilance information	Does a central database exist for managing PV data? *Yes/No, Request documentation to verify.*			
		Does the central database contain data from various PV sources and methods (including PHPs? *Yes/No* *Request documentation to verify.*			
		Is there a dedicated computer for pharmacovigilance activities? *Yes/No*			
		Does the computer have internet access? *Yes/No*			
		Is data stored on a cloud/server? *Yes/No, please specify*			
		Is there a back-up system? *Yes/No, please specify*			
3.2	Evidence of a process or mechanism for sharing information with other regulatory functions, other regulatory agencies and global databases	Has information in the database been shared (either electronically or via report) with other regulatory functions, other regulatory agencies and/or global databases? *Request documentation to verify.*			
3.3	Existence of a standard adverse event (AE) reporting form	Is there a standard AE reporting form? *Request documentation to verify.*			
		How is the reporting form offered? (e.*g.* paperform, web, app)			
	Subset indicators: The standard reporting form, or separate forms, provide for reporting of—	Please report if the following items are included and if it is on the standard AE reporting form or a separate form:			
	- Adverse drug reactions	Are there relevant fields in the standard AE form (or a separate form) to report adverse drug reactions?			
	- Suspected medication errors	Are there relevant fields in the standard AE form (or a separate form) to report suspected medication errors?			
	- Therapeutic ineffectiveness	Are there relevant fields in the standard AE form (or a separate form) to report therapeutic ineffectiveness?			
	- Suspected misuse, abuse of and/or dependence on medicines	Are there relevant fields in the standard AE form (or a separate form) to report suspected misuse, abuse and/or dependence on medicines?			
	- Adverse events following immunization (AEFI)	Are there relevant fields in the standard AE form (or a separate form) to report adverse events following immunization?			
	- Medical devices and diagnostics	Is there a form with relevant fields for reporting suspected/ observed issues with medical devices and diagnostics?			
	- Suspected product quality issues	Is there a form with relevant fields for reporting suspected/ observed poor quality issues?			

Indicator #	Indicator	Assessment Questions	Answer	Notes & additional comments	Information source
3.4	Existence of a form or mechanism for the public to report AEs (Patient reporting system)	Is there a standard reporting form for the general public to report AEs? *Request documentation to verify.*			
3.5	Existence of electronic AE reporting system that complies with international reporting format standards	Is there an electronic AE reporting system? *If yes, please provide technical details.*			
		Is the system compliant with the international reporting standards (E2B)?			
3.6	A process is in place for collection, recording and analysis of ADR reports	Is there a process in place for collection, recording and analysis of ADR reports?			
**Component 4. Risk Assessment and Evaluation**					
4.1	Number of registered products with a PV plan and/or a risk management strategy	How many registered products with a pharmacovigilance plan and/or a risk management strategy from market authorization holders exist in the country?			
4.2	Total number of AE reports received in the previous calendar year (also expressed as number of AEs per 100 000 persons in the population)<br>Sub-indicators:	What is the total number of AE reports received in the previous calendar year? *Request documentation to verify.*			
	- ADR	Of the total, what is the number of reports of ADR?			
	- Suspected medication errors	Of the total, what is the number of reports of suspected medication errors?			
	- Therapeutic ineffectiveness	Of the total, what is the number of reports of therapeutic ineffectiveness?			
	- AEFI	Of the total, what is the number of reports of AEFI?			
	- Suspected misuse, abuse, dependence	Of the total, what is the number of reports of suspected misuse, abuse, dependence?			
	- Suspected falsified / substandard drugs or quality defects	Of the total, what is the number of reports of suspected counterfeit / substandard drugs?			
	- AE/incidents relating to medical devices and diagnostics	Of the total, what is the number of reports of AE related to medical devices and diagnostics?			
		What is the total population of the country?			
4.3	Number and percentage of total AE reports received by the national pharmacovigilance center in the previous calendar year from:				

Indicator #	Indicator	Assessment Questions	Answer	Notes & additional comments	Information source
	- Marketing Authorization Holders	What is the number of AE reports received by the national pharmacovigilance center in the previous calendar year from marketing authorization holders?			
	- PHPs	What is the number of AE reports received by the national pharmacovigilance center in the previous calendar year from public health programs?			
	- Health care providers	What is the number of AE reports received by the national pharmacovigilance center in the previous calendar year from healthcare providers?			
	- Patients	What is the number of AE reports received by the national pharmacovigilance center in the previous calendar year from patients?			
	-Distributors	What is the number of AE reports received by the national pharmacovigilance center in the previous calendar year from distributors?			
	-Suppliers	What is the number of AE reports received by the national pharmacovigilance center in the previous calendar year from suppliers?			
4.4	Number and percentage of total AE reports received that are entered in the national database in the previous calendar year	What is the total number of AE reports received that have been entered in the national database in the previous calendar year?			
4.5	Number and percentage of total AE reports acknowledged and/or issued feedback in the previous calendar year	What is the total number of AE reports acknowledged/issued feedback in the previous 12 months?			
4.6	Number and percentage of AE reports subjected to causality assessment in the previous calendar year	What is the total number of AE reports subjected to causality assessment in the previous calendar year?			
4.7	Number and percentage of AE reports submitted to VigiBase in the previous calendar year	How many of the AE reports received at the national pharmacovigilance center were submitted to Vigibase in the previous calendar year?			
4.8	Average completeness score of quarterly reports submitted to VigiBase in the previous four quarters (= one year)	What was the average completeness score of quarterly reports submitted to Vigibase in the previous calendar year? *Consult quarterly reports from VigiGrade for completeness scores of submitted reports*			
4.9	Number of active surveillance activities initiated, ongoing or completed during the previous three years	How many active surveillance studies have been conducted in the last three years (36 months)?			
		Indicate what type (e.g. cohort event monitoring, targeted spontaneous reporting, etc.) and stage of completion (e.g. initiated, on-going or completed) for each study. *Request documentation to verify. Request research protocol*			

Indicator #	Indicator	Assessment Questions	Answer	Notes & additional comments	Information source
4.10	Number and percentage of total AE reports received at the national pharmacovigilance center in the previous calendar year from healthcare providers by type of provider	What is the number of AE reports received in the previous calendar year submitted by **doctors**?			
		What is the number of AE reports received in the previous calendar year submitted by **nurses or midwifes**?			
		What is the number of AE reports received in the previous calendar year submitted by **pharmacists**?			
		What is the number of AE reports received in the previous calendar year submitted by **manufacturers and pharmaceutical companies**?			
		What is the number of AE reports received in the previous calendar year submitted by **dentists**?			
		What is the number of AE reports received in the previous calendar year submitted by the **general public**?			
		What is the total number of AE reports received in the previous calendar year?			
4.11	Evidence of supervision visits to marketing authorization holders by NMRA that address PV	Does the NMRA conduct supervision visits of MAHs that address PV?			
		How many supervision visits have been conducted in the previous calendar year?			
**Component 5. Risk Management and Communication**					
5.1	Number of regulatory actions taken in the previous calendar year as a consequence of national pharmacovigilance activities including:	*Request documentation to verify.*			
	- Number of product label changes (variation);	How many regulatory actions were taken in the previous calendar year as a consequence of pharmacovigilance activities that resulted in *product label changes (variation)*?			
	- Number of safety warnings on medicines to health professionals and general public;	How many regulatory actions were taken in the previous calendar year as a consequence of pharmacovigilance activities that resulted in *safety warnings on medicines to health professionals*?			
		How many regulatory actions were taken in the previous calendar year as a consequence of pharmacovigilance activities that resulted in *safety warnings on medicines to the general public*?			
	- Number of withdrawals of medicines;	How many regulatory actions were taken in the previous calendar year as a consequence of pharmacovigilance activities that resulted in *withdrawals of medicines*?			
	- Number of other restrictions on use of medicines;	How many regulatory actions were taken in the previous calendar year as a consequence of pharmacovigilance activities that resulted in *other restrictions on use of medicines*?			
	- Number of treatment guideline/policy changes	How many regulatory actions were taken in the previous calendar year as a consequence of pharmacovigilance activities that resulted in *treatment guideline/policy changes*?			

Indicator #	Indicator	Assessment Questions	Answer	Notes & additional comments	Information source
5.2	Number of signals detected in the past 5 years by the pharmacovigilance center	How many signals were detected in the past 5 years by the pharmacovigilance center?			
		If any signals were detected, which ones and how were they identified?			
5.3	Average time lag between identification of safety signal of a serious ADR or significant medicine safety issue generated nationally and communication to health care workers and the public	How long does it take from when a safety signal or significant safety issue is identified to when it is communicated to health workers and the public? *Please answer in days for each signal identified in the previous calendar year.*			
5.4	Number of suspected product quality issues detected through the pharmacovigilance system	What is the number of suspected product quality issues detected through the pharmacovigilance system in the previous calendar year? *Request documentation to verify.*			
5.5	Percentage of planned issues of the medicine safety bulletin (or any other health-related newsletter that routinely features ADR or medicine safety issues) published in the previous 12 months	How many issues of the medicine safety bulletin are supposed to be published per year?			
		How many issues of the medicine safety bulletin were published in the previous 12 months? *Request documentation to verify.*			
5.6	Number of products voluntarily withdrawn by marketing authorization holders because of safety concerns in the previous calendar year	How many products were voluntarily withdrawn by marketing authorization holders because of safety concerns in the past 12 months? *Request documentation to verify.*			
5.7	Number and percentage of medicine safety information requests addressed in the previous 12 months	How many requests for information about medicine safety were received in the previous 12 months? *Request documentation to verify.*			
		Of the total received, how many requests for medicine safety information were addressed in the previous 12 months?			
5.8	Number of summaries of product characteristics updated by MAH because of safety concerns in the previous year	Of the total received, how many requests for medicine safety information were addressed in the previous 12 months?			
5.9	Number of medicine safety issues of local relevance identified from outside sources (e.g., from another country, from EAC region or international sources) and acted on locally in the previous calendar year	How many medicine safety issues identified from outside sources were acted on locally in the previous calendar year? *Request documentation to verify.*			

Indicator #	Indicator	Assessment Questions	Answer	Notes & additional comments	Information source
5.10	Number of public or community education activities relating to medicine safety carried out in the previous calendar year	How many public or community education activities relating to medicine safety were carried out in the previous calendar year? *Request documentation to verify.*			

Indicator #	Indicator	Assessment Questions	Answer	Notes & additional comments	Information source
**Component 2. Systems, Structures, and Stakeholder Coordination**					
P2.1	Pharmacovigilance activities included within the strategic and/or annual operational plans of public health programs	Are pharmacovigilance activities included within the strategic and/or annual operational plans of public health programs? *Yes/No Request documentation to verify.*			
P2.2	Existence of a dedicated financial provision or statutory budget for the PHPs	Is there an annual budgetary allocation for pharmacovigilance activities for the PHP? *Yes/No Request documentation to verify.*			
		In the last fiscal year, how many funds were allocated by the MOH and donors for pharmacovigilance activities? *Please enter the amount in the Answer box and specify the currency in the Notes column.*			
P2.3	Existence of a mechanism to disseminate pharmacovigilance information (including one or more of the following: newsletters, information bulletin, website or phone line for dissemination of pharmacovigilance information)	Is there a mechanism in place to disseminate PV information? *Yes/No Request documentation to verify.*			
		Is there a newsletter or information bulletin for dissemination of PV information? *Request documentation to verify.*			
		Is there a website for dissemination of PV information? *Yes/No Request documentation to verify.*			
		Is there a publicly advertised phone line to receive and provide medicine safety and PV information? *Yes/No Request documentation to verify.*			
		Is there another mechanism for dissemination of PV information? *Please describe the mechanism*			
P2.4	Number of healthcare workers trained in pharmacovigilance in the previous calendar year through in-service training	How many healthcare workers has the center/program trained on PV in the previous calendar year (through **in-service** training)? *Request documentation to verify.*<br>- Clinicians / nurses			
		- Community health workers			
		How many training events/sessions were conducted in the previous calendar year? *Request documentation to verify.*			
P2.5	Number of national treatment guidelines or protocols in use within the public health programs that consider pharmacovigilance	Do the treatment guidelines or protocols in use in the PHP provide instruction for PV activities? *Yes/No Request documentation to verify.*			
P2.6	Evidence of consideration of safety data when developing and updating standard treatment guidelines or treatment policies	When developing standard treatment guidelines is evidence on safety data being described and taken into consideration? *Yes/No Request documentation to verify.*			
**Component 3. Signal Generation and Data Management**					
P3.1	PHPs use the national, standard ADR/AE reporting form	Does the PHP use the national, standard ADR/AE reporting form? *Yes/No*<br>*Request a copy of all existing reporting forms.*			

Indicator #	Indicator	Assessment Questions	Answer	Notes & additional comments	Information source
**Component 4. Risk Assessment and Evaluation**					
P4.1	Number and percentage of ADR/AE reports received by PHPs that were submitted to the national pharmacovigilance center in the previous calendar year	What is the number of AE reports received by the PHP in the previous calendar year?			
		What is the number of AE reports submitted by the PHP to the national PV center in the previous calendar year?			
P4.2	Total number of ADR reports per 1000 individuals exposed to medicines in the PHP in the previous calendar year.	How many individuals received medicines under the PHP in question during the previous year?			
		How many ADR reports were received, referring to the exposed population?			
P4.3	Percentage of patients in public health programs for whom drug-related, serious unexpected/unknown adverse events were reported in the previous calendar year	What is the total number of patients receiving medicines under the PHP? *Request documentation to verify.*			
		What is the total number of patients receiving medicines in the PHP who experienced drug-related, serious, unexpected adverse events? *Request documentation to verify.*			
		How many of those were reported to the national PV center? *Request documentation to verify.*			
P4.4	Number of suspected product quality issues detected through public health programs	What is the number of suspected product quality issues detected through the PHP in the previous calendar year?			
P4.5	Number of reports on therapeutic ineffectiveness in the previous year	What is the number of reports on therapeutic ineffectiveness received by the PHP in the previous calendar year?			
P4.6	Number of medicine-related hospital admissions per 1000 individuals exposed to medicines in the PHP in the previous year	What is the number of medicine-related hospital admissions of individuals exposed to medicines in the PHP in the previous year?			
P4.7	Number of active surveillance activities initiated, ongoing or completed during the past three years	How many active surveillance studies have been conducted in the last three years (36 months)?			
		Indicate what type (e.g. cohort event monitoring, targeted spontaneous reporting, etc.) and stage of completion (e.g. initiated, on-going or completed) for each study *Request documentation to verify*			
P4.8	Functional collaboration/involvement in risk management plans with the PV centre	Do the PHP and PV centre communicate on risk management plans?			
		How often have the PHP and PV center met to discuss risk management in the previous calendar year? *Request documentation to verify.*			

Indicator #	Indicator	Assessment Questions	Answer	Notes & additional comments	Information source
**Component 5. Risk Management and Communication**					
P5.1	Average time lag between identification of safety signal of a serious ADR or significant medicine safety issue generated nationally and communication to health care workers and the public	How long does it take from when a safety signal or significant safety issue is identified to when it is communicated to health workers and the public? *Please enter your answer in days for each signal.*			
P5.2	Existence of a program-related newsletter that routinely features ADR or medicine safety information	Is there a program-related newsletter, bulletin or other publication that routinely features ADR or medicine safety information?			
P5.3	Number and percentage of medicine safety information requests addressed in the previous calendar year	How many requests for information about medicine safety were received in the previous calendar year? *Request documentation to verify.*			
		How many requests for medicine safety information were addressed in the previous calendar year? *Request documentation to verify.*			
P5.4	Number of medicine safety issues of local relevance identified from outside sources (e.g., from another country, from EAC region or international sources) and acted on locally in the previous calendar year	How many medicine safety issues identified from outside sources were acted on locally in the previous calendar year? *Request documentation to verify.*			
P5.5	Number of public or community education activities relating to medicine safety carried out in the previous calendar year	How many public or community education activities relating to medicine safety were carried out by the PHP in the previous calendar year? *Request documentation to verify.*			
