# Supplementary material for: Baseline assessment of pharmacovigilance activities in four sub-Saharan African countries: a perspective on tuberculosis
Source: BMC Health Serv Res. 2021 Oct 8;21:1062. doi: 10.1186/s12913-021-07043-6 (PMC8499544; doi:10.1186/s12913-021-07043-6)
Supplement: Supplementary file 2 — Additional file 2. [file 12913_2021_7043_MOESM2_ESM.pdf]

PAVIA / PROFORMA topics for group interviews

The aim of the interviews is to get a good overview of the current situation of the pharmacovigilance (PV) system in the country and to identify barriers and enablers to improve the PV system. The baseline indicators (based on the indicator list developed by EAC which has already been used by TFDA in Tanzania and 2 other east-Africa countries) will be the starting point for the interviews. Depending on the reactions of the interviewees additional questions may be raised to seek clarification (see Examples).

Topics include

- Policy, law and regulation regarding pharmacovigilance
- Systems, structures and stakeholder coordination
- Signal generation and data management
- Risk assessment and evaluation
- Risk management and communication
- Public health programs (PHP), specifically TB and mass-drug administration including immunization; same items as mentioned above
- Reporting attitudes, practice and experience by health care workers
- Pharmacovigilance in formal education

Examples

Indicator 1.2 (WHO CST2, IPAT 1.1)

Are there legal provisions for pharmacovigilance or medicine safety in the medicines act or law?

(i) If yes, when introduced ____;
(ii) How was it developed?
(iii) What was the role of Parliament regarding the legislation? Was it discussed in and approved by the parliament?
(iv) Are the legal provisions executed and enforced?
What demonstrates that?
(v) If no, what are the reasons?
(vi) To what extent has it been implemented considering strategies and targets?
(vii) What have been challenges in the implementation phase?
(viii) When last updated/revised_____
(ix) If no legislation available, what are the challenges

Indicator 2.4 (WHO CST10, IPAT 2.6)

Does a national medicine safety advisory committee exist with the responsibility to provide technical advice on the safety of medicines to the regulatory authority?

i) What is the relation between the medicine safety advisory committee and the general drug regulatory authority (if present)?
ii) How many members does the medicine safety advisory committee have and what are their competencies/qualifications?
iii) Did the members sign a confidentiality agreement?

Indicator P4.7 (WHO CP9, IPAT 4.5)

How many active surveillance studies have been conducted in the last three years (36 months)?
Indicate what type (e.g. cohort event monitoring, targeted spontaneous reporting, etc.) and stage of completion (e.g. initiated, on-going or completed) for each study.

a. What were facilitators/barriers in the implementation of the study?
b. How was data analysis done?
c. What new knowledge has the study given?
d. In case of new knowledge, has this been used in any harm-benefit decision making?

Specific questions to be included re. the National TB program:

For the NTP focal persons:

- In how many centers is MDR TB treated?
- How many centers use new regimens? (short treatment, bedaquilline or delaminid)
- Which aDSM package is used?
- Which treatment regimens are used in country?
- Which TB treatment centers use aDSM?
- For which patients is aDSM done? (only new drugs and regimens? All MDR TB? All TB patients?)
- How is the collaboration with the PV unit?
  - Who is responsible for what activities?
  - How often do PV unit staff and NTP staff meet?
  - Any hurdles in collaboration, and how were these solved?
- Regarding causality assessment of AE and signal detection, who holds final responsibility for what, what goes well and what not? What could be improved?
- Is feedback being provided to the reporters? If yes, what type of feedback (request documentation): acknowledgement of receipt only, specific comments/follow-up from the report, or more general?
- Do you do joint monitoring visits of health centers together with PV center, why (not)? If yes: do you see added value of doing the visits together? Why (not)?
- How do you experience aDSM? Does it help to improve patient care? Is it feasible regarding workload for health care workers, and for NTP staff in terms of monitoring the data collection?

Health facility

- Has staff been trained on PV / aDSM? By whom?
- Is equipment (*e.g.* audiometry, ECG machine) in place to monitor for possible AE and is it functioning? Have any problems with the equipment occurred and how did you solve these?
- Are ancillary drugs available free of charge to patients who need it?
- Are AE/ADR reporting forms easily accessible for staff?
- Are AE/ADR reporting forms electronically or paper-based?
- Do you receive feedback on reports? If yes: what type of feedback? (acknowledgement of receipt only, specific comments/follow-up from the report, or more general) – If available: ask for the documentation.
- Are AEs that require treatment change being discussed amongst MDR-TB experts/consilium before treatment change is made?
- How many health care workers have reported an AE in the previous calendar year?
- How many reports have been submitted by the health facility? (calculate reports / patients on TB treatment)

HCW (doctor, nurse, pharmacist)

- How often did you think of AE in last year?
- Do you report ***all*** AEs requiring medical intervention that are recorded in the patient file? If not: why not?
- How often did you report an AE in the last year?
- If you did not report all suspected AE, what was the reason?
- When would you report an AE? (*e.g.* all AE or only specific ones? Only when sure it is caused by a certain drug?)
- What did you do with the AE? (stop treatment, treat side-effect)
- How do you experience aDSM? (What are advantages and disadvantages to your opinion? Does it improve patient care? Is it beneficial? Is it feasible regarding workload?)
- Are reporting forms easily accessible?
- What are barriers for reporting AE?
- What would facilitate reporting of AE?
- Do you receive feedback on reports?
